# Identification and validation of a novel risk model based on cuproptosis‑associated m6A for head and neck squamous cell carcinoma

**DOI:** 10.1186/s12920-024-01916-5

**Published:** 2024-05-22

**Authors:** Zhongxu Xing, Yijun Xu, Xiaoyan Xu, Kaiwen Yang, Songbing Qin, Yang Jiao, Lili Wang

**Affiliations:** 1 Department of Radiation Oncology, The First Affiliated Hospital of Soochow University, Suzhou, 21500 China; 2 State Key Laboratory of Radiation Medicine and Protection, School of Radiation Medicine and Protection, Collaborative Innovation Center of Radiological Medicine of Jiangsu Higher Education Institutions, Soochow University, Suzhou, 215123 China

**Keywords:** Head and neck squamous cell carcinoma, RNA methylation regulation, Cuproptosis, Prognosis, Risk model

## Abstract

**Background:**

Head and neck squamous cell carcinoma (HNSCC) is a prevalent cancer with a poor survival rate due to anatomical limitations of the head and a lack of reliable biomarkers. Cuproptosis represents a novel cellular regulated death pathway, and N6-methyladenosine (m6A) is the most common internal RNA modification in mRNA. They are intricately connected to tumor formation, progression, and prognosis. This study aimed to construct a risk model for HNSCC using a set of mRNAs associated with m6A regulators and cuproptosis genes (mcrmRNA).

**Methods:**

RNA-seq and clinical data of HNSCC patients from The Cancer Genome Atlas (TCGA) database were analyzed to develop a risk model through the least absolute shrinkage and selection operator (LASSO) analysis. Survival analysis and receiver operating characteristic (ROC) analysis were performed for the high- and low-risk groups. Additionally, the model was validated using the GSE41613 dataset from the Gene Expression Omnibus (GEO) database. GSEA and CIBERSORT were applied to investigate the immune microenvironment of HNSCC.

**Results:**

A risk model consisting of 32 mcrmRNA was developed using the LASSO analysis. The risk score of patients was confirmed to be an independent prognostic indicator by multivariate Cox analysis. The high-risk group exhibited a higher tumor mutation burden. Additionally, CIBERSORT analysis indicated varying levels of immune cell infiltration between the two groups. Significant disparities in drug sensitivity to common medications were also observed. Enrichment analysis further unveiled significant differences in metabolic pathways and RNA processing between the two groups.

**Conclusions:**

Our risk model can predict outcomes for HNSCC patients and offers valuable insights for personalized therapeutic approaches.

## Background

Head and neck squamous cell carcinoma (HNSCC) is a prevalent malignancy on a global scale [1]. The prevalence of HNSCC is rising, with an anticipated surge of 30% by 2030 [2]. Despite advancements in various therapeutic strategies, the 5-year overall survival rate for patients with HNSCC remains below 50% [3]. In recent years, the construction of biomarker-based prognostic models for cancer has received increasing attention [4–6]. However, the prognostic predictive results still lack high accuracy due to the limitation of relying solely on a single biomarker. The integration of two biomarkers enhances the accuracy of the prognostic model [7]. Increasing evidence supports results derived from prognostic models constructed with the integration of two biomarkers [8, 9]. Incorporating additional biomarkers is deemed necessary to construct more accurate prognostic models.

Cuproptosis is a distinctive form of copper-induced programmed cell death discovered by Tsvetkov in 2022 and follows distinct mechanisms independent of conventional cell death pathways. Copper is an essential trace element in many biological processes. However, excess copper binds directly to lipoylated proteins in the tricarboxylic acid (TCA) cycle, leading to the aggregation of lipoylated proteins and subsequent loss of iron-sulfur clusters. This process induces proteotoxic stress and ultimately triggers cuproptosis [10]. Additionally, cuproptosis-related signatures have shown the ability to predict the prognosis and immune response in various types of cancers, including bladder cancer and breast cancer [11, 12]. N6-methyladenosine (m6A) represents one of the most prevalent internal RNA modifications, playing a vital role in various cellular processes such as RNA synthesis, transport, and translation [13]. M6A exhibits promising potential in regulating cell proliferation and cancer immunity in tumors [14, 15], serving as a crucial indicator [16]. Notably, there exists a close association between m6A and various modes of programmed cell death [17, 18]. However, studies on cuproptosis-associated m6A in HNSCC are limited. Further studies into the role of cuproptosis and m6A in HNSCC are imperative.

In this study, we conducted comprehensive bioinformatic analyses and identified a close relationship between mRNAs associated with m6A regulators and cuproptosis genes, with implications for the survival outcomes and immune landscape of HNSCC patients. Hence, we aimed to construct a prognostic model for HNSCC based on mRNA related to m6A and cuproptosis (mcrmRNA).

## Materials and methods

### Data extraction and processing

**Fig. 1:**
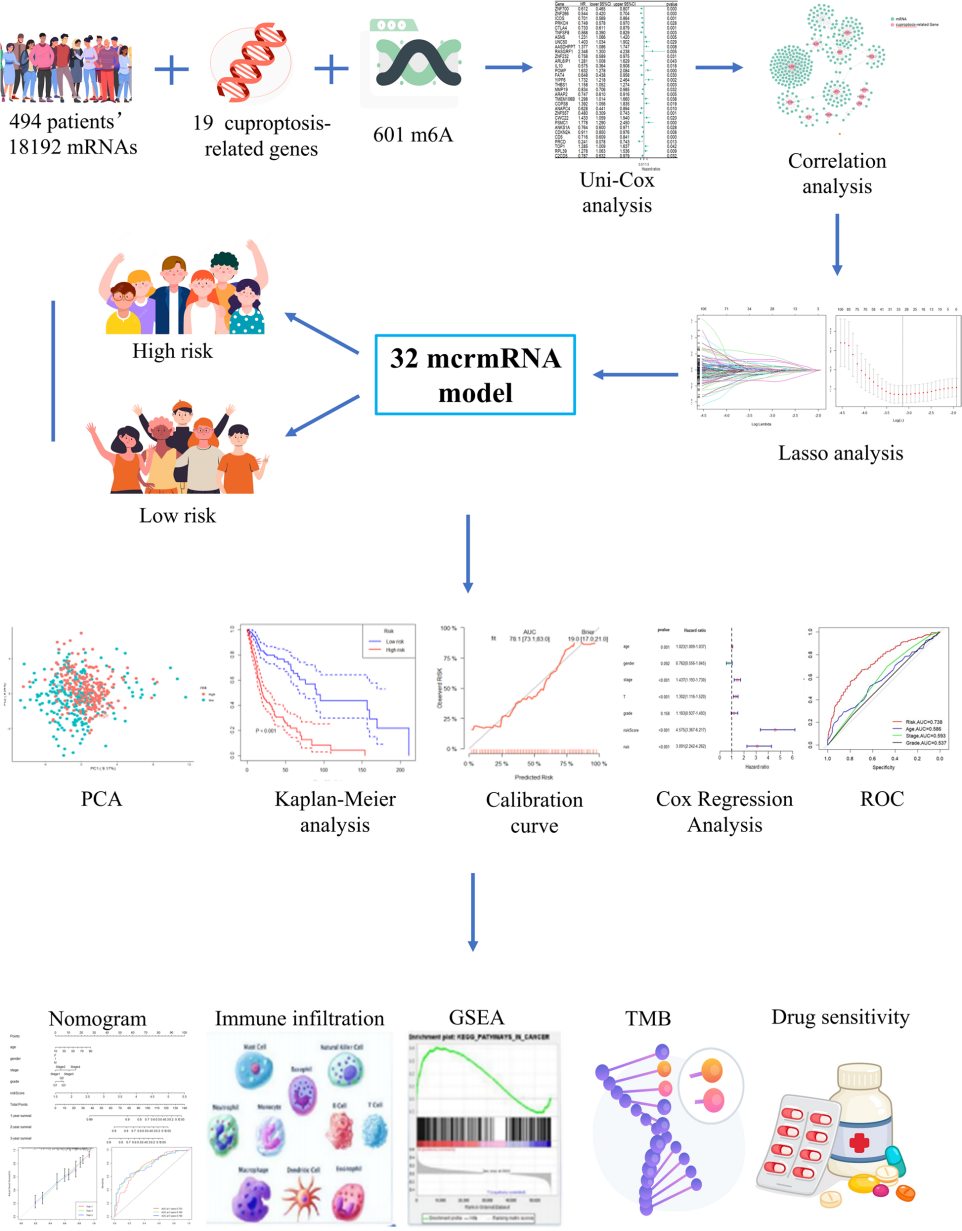
Flowchart

Figure 1 illustrates the research process of this study. We obtained 494 data files containing HNSCC transcriptome data and clinical characteristics data from the TCGA database (TCGA, https://portal.gdc.cancer.gov/). Additionally, we acquired the GSE41613 dataset, comprising 97 HNSCC patients from the Gene Expression Omnibus (GEO) database (https://www.ncbi.nlm.nih.gov/geo/). Patients with missing clinical information, such as survival data, were excluded from the analysis. Ensembl IDs were converted to official gene symbols using the R language (version 4.2.1, https://www.rproject.org/) and the “limma” package. The RNA was then categorized, resulting in 18,192 mRNAs.

### Screening of mcrmRNA

First, a univariate Cox regression analysis was conducted on the mRNA gene data from a cohort of 494 patients, resulting in the identification of 2,721 genes that exhibited significant associations with prognosis. Subsequently, 19 genes related to cuproptosis were extracted from the latest literature [11], while 602 m6A regulators were retrieved from the RM2Target database (http://rm2target.canceromics.org/). Spearman correlation analysis was then performed, with a correlation coefficient threshold of *Cor* > 0.50 and a significance level of *p* < 0.001, to explore the relationships between the prognosis-related genes and cuproptosis-related genes and m6A regulators, respectively. The intersection of these analyses yielded a distinct set of mRNA molecules (referred to as mcrmRNA) that were significantly associated not only with prognosis but also with both m6A regulators and cuproptosis. Finally, the co-expression network of these identified genes was constructed using Cytoscape software (version 3.9.0).

### Construction of the mcrmRNA prognostic risk model

A cohort of 494 patients with HNSCC was randomly allocated into a training set and a testing set at a ratio of 2:1. The training set comprised 329 patients, while the testing set consisted of 165 patients. In the training set, the LASSO Cox regression method was performed using the “glmnet” R package to identify the prognostic model based on the expression of mcrmRNA. Specifically, we utilized tenfold cross-validation to select the optimal lambda value. During each fold of cross-validation, the lambda values were tuned to maximize the model's performance metrics. The lambda value that yielded the best average performance across all folds was chosen for the final model. Subsequently, 32 mcrmRNA were selected for developing the prognostic risk model. The risk score for each HNSCC patient was calculated using the following equation: Risk score = Coefi mcrmRNA1 × mcrmRNA1 expression + Coefi mcrmRNA2 × mcrmRNA2 expression + … + Coefi mcrmRNAn × mcrmRNAn expression.

### Validation of the mcrmRNA prognostic risk model

The patients in the training set were categorized into high- and low-risk groups based on the median risk score. Risk score distribution plots were then generated to visualize the distribution of risk scores. In addition, a comparison was conducted between high- and low-risk groups in terms of survival time and status.

Principal Component Analysis (PCA) is a commonly employed technique for downscaling and feature extraction, facilitating the investigation of disparities within these subgroups. The “ggplot2” package in the R language was utilized to independently conduct PCA analysis on the training set, testing set, and the entire patient population to investigate potential differences between high- and low-risk groups.

Subsequently, Kaplan–Meier analysis was performed between high- and low-risk patients to establish the risk score as an independent prognostic indicator in clinical practice. Calibration plots of risk scores for the training set, test set, and entire patient cohort were generated using the “riskRegression” package in the R language to validate the agreement between the risk score produced by the model and the actual clinical outcomes of the patients. We also compared risk scores with other clinical characteristics using univariate and multivariate Cox regression analyses. Additionally, we generate time-dependent receiver operating characteristic (ROC) curves to measure the performance of the risk model.

Utilizing the GSE41613 dataset as the external validation set, this study applied the risk score calculation formula derived from the training cohort. The cases were then divided into low- and high-risk groups based on an optimal threshold. Subsequently, ROC survival analysis was conducted on the validation set to evaluate the predictive accuracy of the risk model.

### Construction of the nomogram

A nomogram was constructed using the “rms” package to incorporate clinical factors and the risk model. It was then utilized to assess the 1-, 3-, and 5-year survival rates of HNSCC patients. The consistency between the expected and observed survival rates was verified through calibration curves. Additionally, the predictive value of the nomogram was evaluated using time-dependent ROC curves.

### Immune infiltrate analysis

The proportion of 22 immune cells between high- and low-risk groups was estimated using CIBERSORT to assess immune infiltration. We examined differences in immune cells and immune-related functions between high- and low-risk groups using single-sample Gene Set Enrichment Analysis (ssGSEA). The ESTIMATE was utilized to assess tumor purity based on analysis of stromal and immune cells between high- and low-risk groups. Lastly, we explored the expression of immune checkpoint genes in high- and low-risk groups.

### Tumor mutation burden analysis

Furthermore, we retrieved somatic mutation profiles of HNSCC samples from the TCGA somatic mutation database. We analyzed the tumor mutational burden (TMB) of HNSCC samples in high- and low-risk groups, using the “MAFTOOLS” R package.

### Drug sensitivity analysis

The IC50 values of conventional chemotherapeutic drugs in HNSCC were predicted using the “oncoPredict” package in R to identify potential antitumor drugs for the treatment of HNSCC.

### Function enrichment analysis

We conducted Gene Ontology (GO) and Kyoto Encyclopedia of Genes and Genomes (KEGG) pathway analyses respectively, to investigate the molecular mechanisms and biological processes underlying the mcrmRNA prognostic risk model between high- and low-risk groups. Additionally, we selected the annotated gene set file c5.all.v7.0.entrez.gmt and performed Gene Set Enrichment Analysis (GSEA) with a significance level of *p* < 0.05 to identify the top ten important pathways between high- and low-risk groups.

## Results

### Construction of the mcrmRNA prognostic risk model

**Fig. 2:**
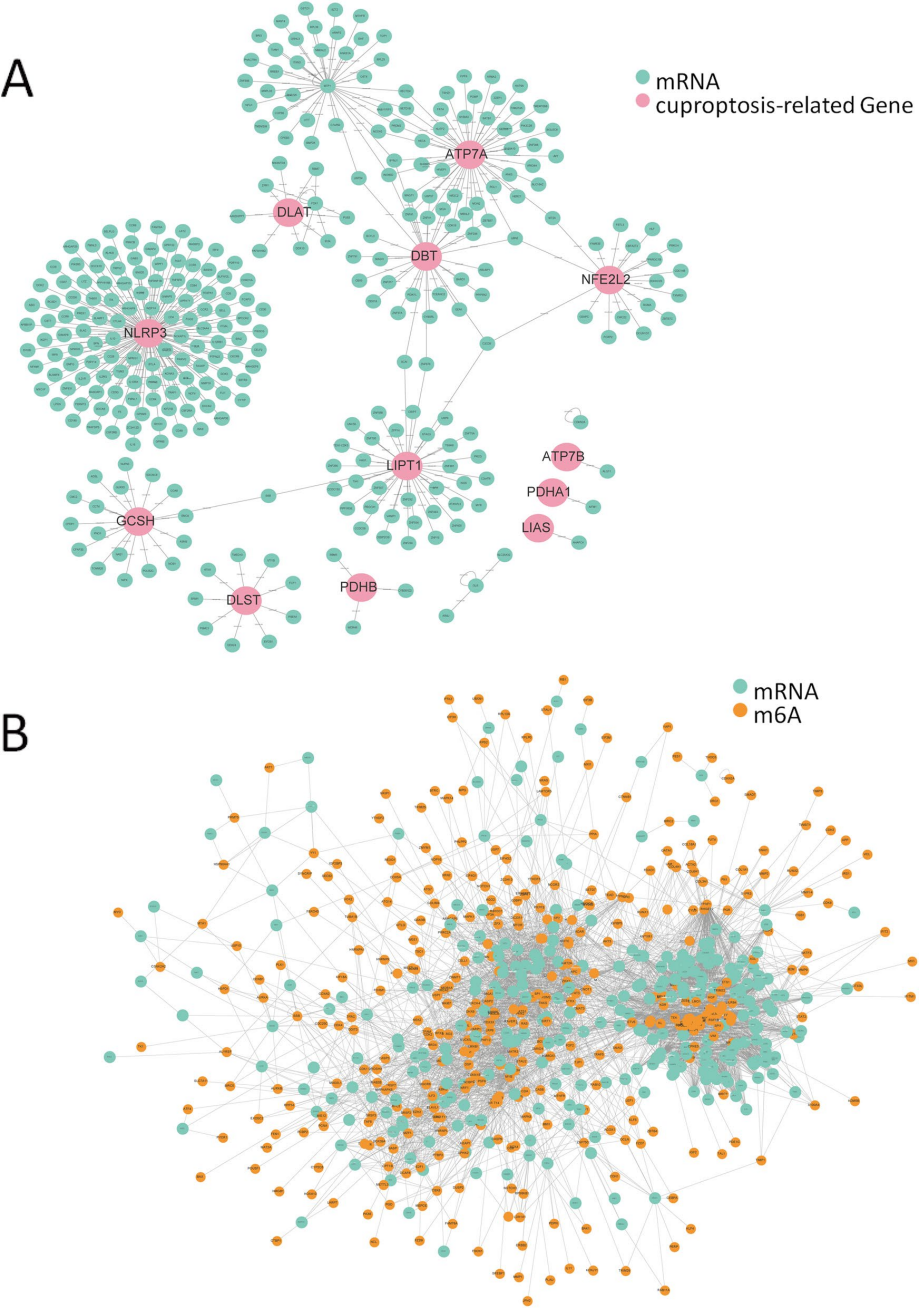
Co-expression network of 12 copper death-related genes and 346 copper death-related prognostic mRNAs (crmRNAs) (*Cor* > 0.5, *p* < 0.001) (**A**). **B** Co-expression network of 601 m6A-related genes and 297 m6A-related prognostic mRNAs (mrmRNAs) (*Cor* > 0.5, *p* < 0.001)

A univariate Cox regression analysis was performed on 494 patients, and 2,721 mRNAs (*p* < 0.05) were found to be significantly associated with patient prognosis. Based on the Pearson co-expression analysis, we ultimately selected 346 prognosis-related mRNAs specific to cuproptosis (crmRNA) and 297 prognosis-related mRNAs specific to m6A regulators (mrmRNA) (Fig. 2A, B). The intersection of these sets yielded 297 prognosis-related mRNA (mcrmRNA), which includes mRNAs associated with prognosis and correlated with m6A regulators and cuproptosis-related genes.

**Fig. 3:**
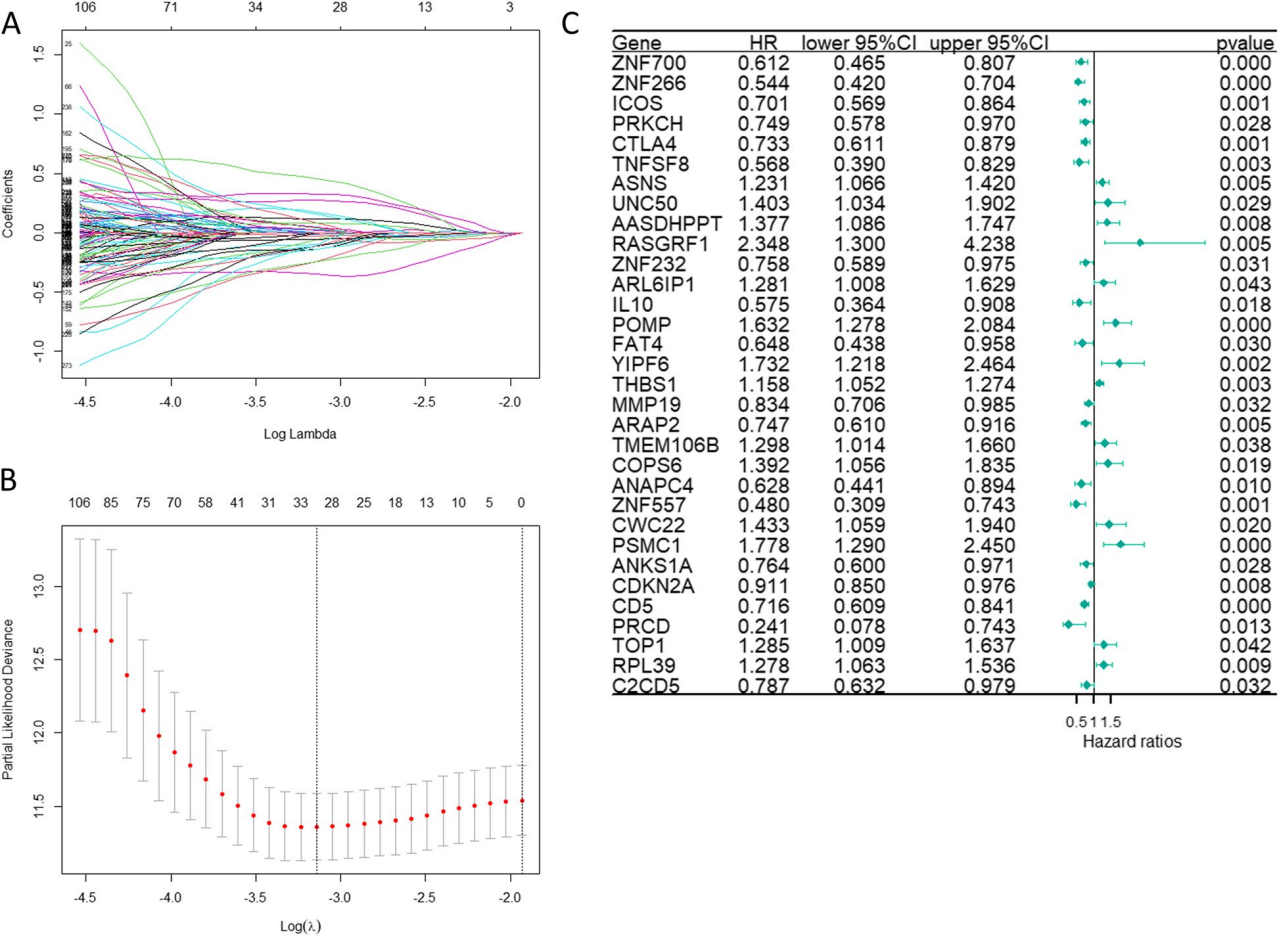
LASSO algorithm analysis identified 32 mcrmRNA for the construction of the prognostic risk model (**A**, **B**). The tuning parameter (lambda) in the LASSO model was selected via tenfold cross-validation. The relationship between the partial likelihood deviation (binomial deviation) and log(lambda) was visualized. Dotted vertical lines were positioned at the optimal lambda values determined by the minimum criteria and the 1 standard error (SE) of the minimum criteria (the 1-SE criteria). A coefficient profile plot was generated against the log(lambda) sequence. A vertical line was drawn at the lambda value selected through tenfold cross-validation, resulting in 32 features with non-zero coefficients under the optimal lambda. **C** The forest plot reveals that the 32 mcrmRNA used in the prognostic risk model has significant prognostic value

**Fig. 4:**
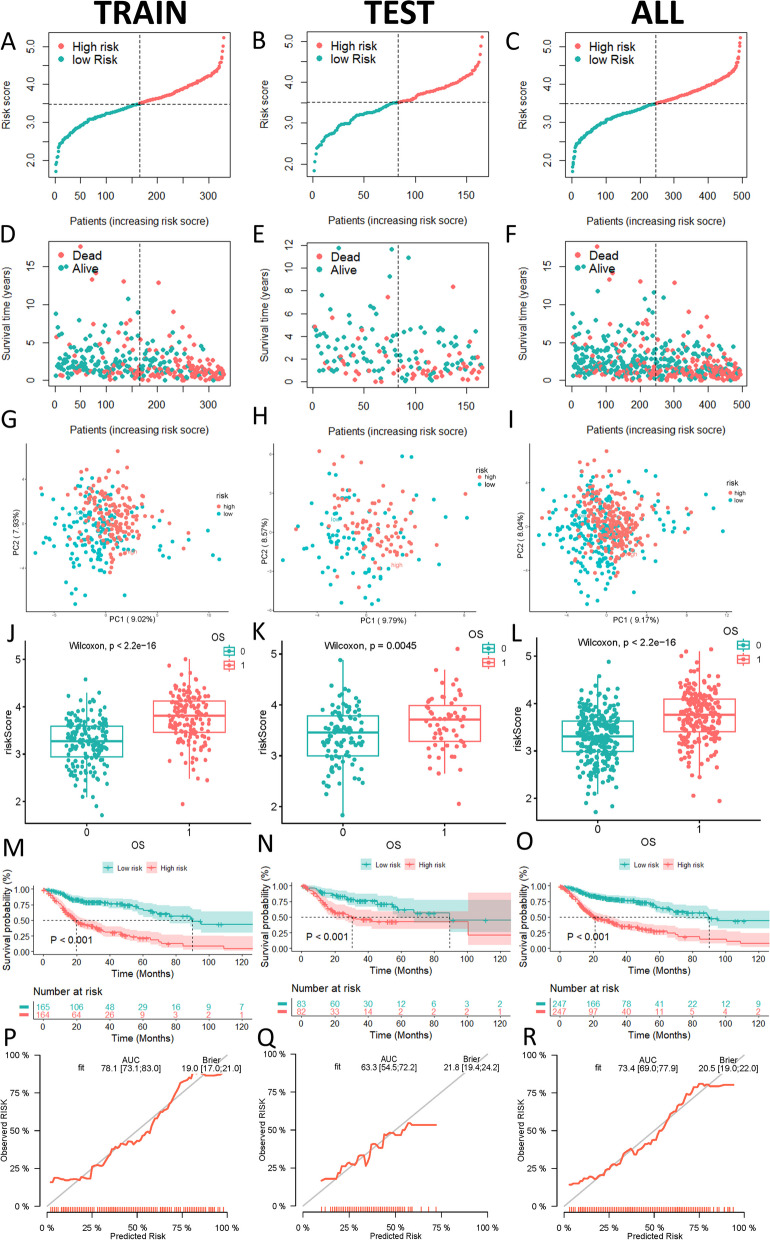
Validation of the prognostic risk model for HNSCC patients based on mcrmRNA using data from TCGA database. Risk score distribution (**A**-**C**), survival status (**D**-**F**), PCA (**G**-**I**), survival status box plots (**J**-**L**), Kaplan–Meier survival analysis for overall survival (OS) in the training, testing, and entire sets for high- and low-risk groups (**M**–**O**), and calibration plots (**P**-**R**)

Following this, 32 prognostic mRNAs were identified in the training set for the construction of the prognostic risk model using LASSO analysis (Fig. 3A, B). Accordingly, we computed the risk score of each patient by calculating the expression of each prognostic mRNA multiplied by its corresponding coefficient. The forest plot illustrates the results of Cox regression analysis for these 32 prognostic mRNAs (*p* < 0.05) (Fig. 3C). Cases in the training set, testing set, and entire cohort were divided into high- and low-risk groups according to the median value of the risk score. The risk distribution, survival status, PCA, calibration plot, and Kaplan–Meier analyses were validated in the training set, testing set, and overall cohort (Fig. 4A-R). The results of the analysis for the three groups had the same trend. Risk curves and scatter plots demonstrated a significant increase in mortality with increasing levels of risk. The results of PCA showed a clear distinction between the high- and low-risk groups. Kaplan–Meier curves revealed a higher survival rate in the low-risk group compared to the high-risk group (*p* < 0.001). Calibration plots indicated area under the curve (AUC) values of the prognostic model were 0.781 in the training set, 0.633 in the testing set, and 0.734 in the entire cohort. These results demonstrated the favorable predictive ability of the prognostic risk model.

**Fig. 5:**
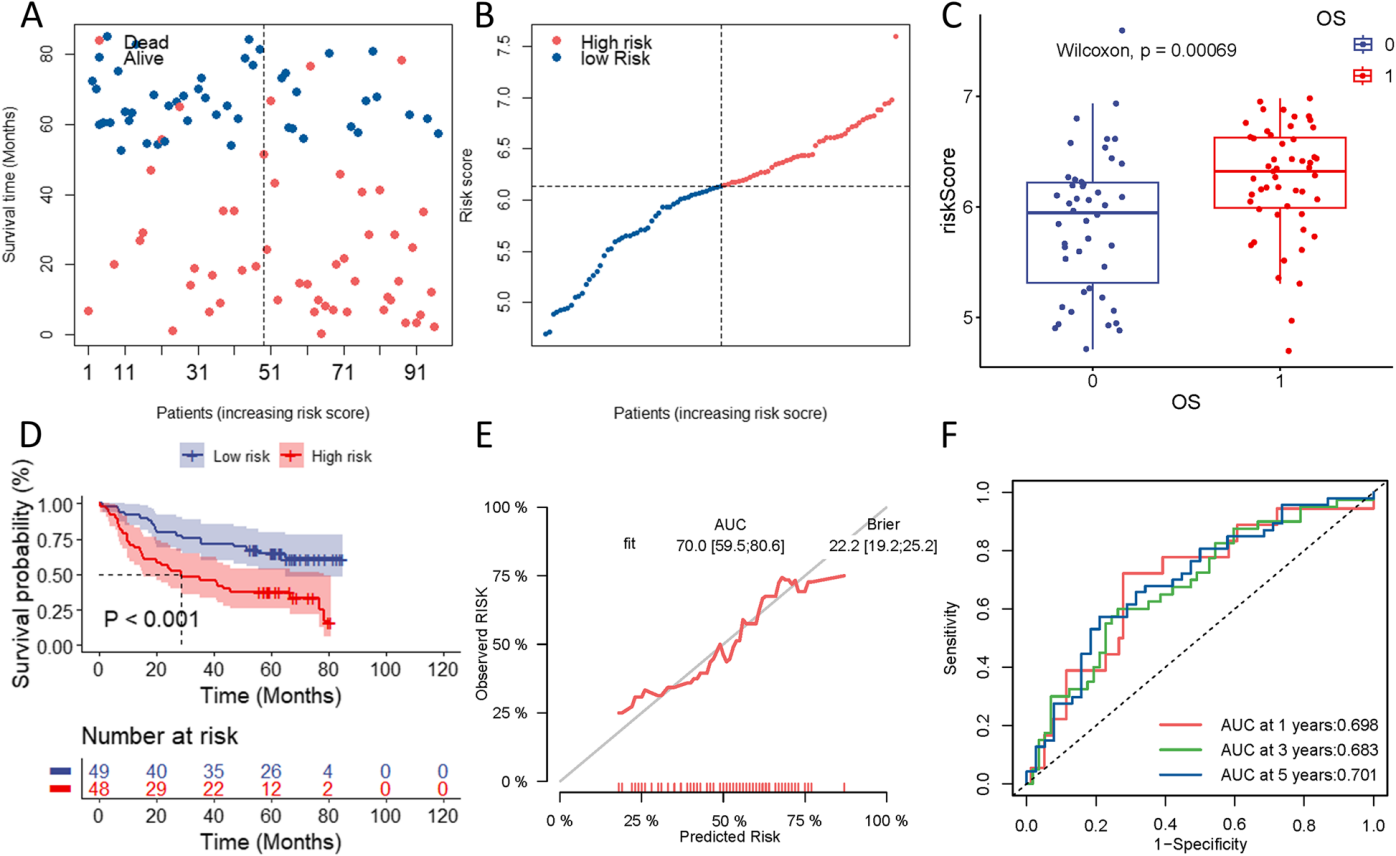
Validation of the prognostic risk model for HNSCC patients using data from the GEO database. The distribution of risk scores among the patient groups categorized as high-risk and low-risk (**A**, **B**). The box plots the survival status of patients across the risk groups (**C**). The Kaplan–Meier curves for survival status and survival time between high-risk and low-risk HNSCC patients (**D**). The calibration plots of the prognostic model (**E**). The time-dependent ROC curve of the risk score (**F**)

To validate the accuracy of our constructed prognostic risk model, we utilized transcriptomic and clinical data of 97 HNSCC patients sourced from the GEO database. The patients were divided into high- and low-risk groups using the same risk calculation formula as applied in the training set, with risk scores based on the median value serving as the threshold. Specifically, 48 patients were categorized as high-risk and 49 as low-risk. Similar to the training set, we observed that higher risk scores were associated with lower survival rates in this validation cohort (Fig. 5A-C). The survival analysis depicted in Fig. 5D revealed a significantly lower survival rate in high-risk cases compared to low-risk cases (*p* < 0.001). Additionally, time-dependent ROC analysis demonstrated the strong predictive performance of our model, with AUC values of 0.698 for 1-year, 0.683 for 3-year, and 0.701 for 5-year survival (Fig. 5F). Calibration plots also supported the reliability of the prognostic model, showing an AUC value of 0.700 (Fig. 5E). These findings collectively underscore the robustness and accuracy of our model in predicting HNSCC prognosis.

### Independent *Indicator* for the mcrmRNA prognostic risk model

**Fig. 6:**
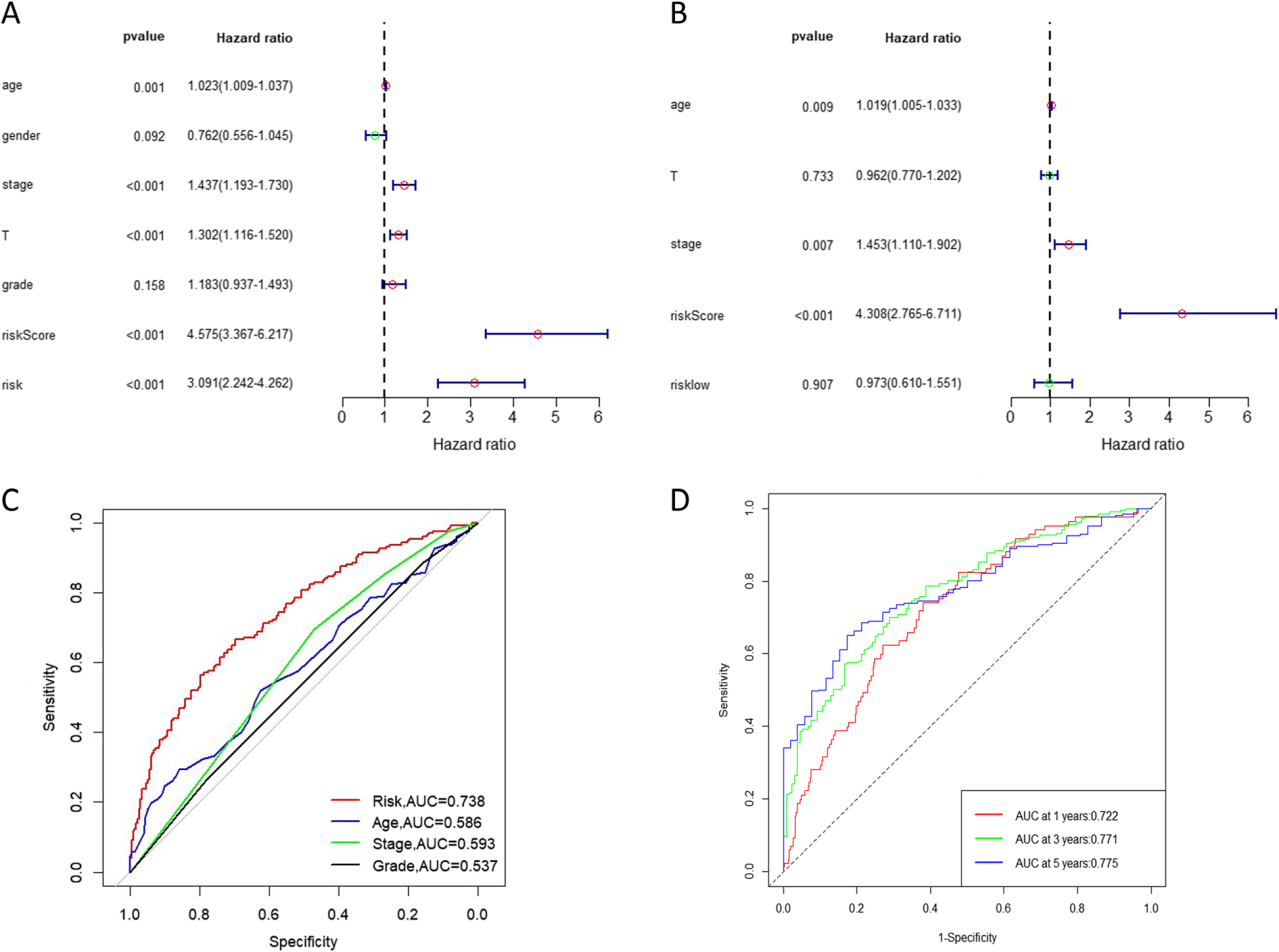
Independent prognostic value of risk score. **A** Univariate and (**B**) Multivariate Cox Regression Analysis results for HNSCC. **C** ROC curve for prognostic indicators in HNSCC. (D) Time-dependent ROC curve for HNSCC risk score

To determine whether the prognostic risk model based on mcrmRNA is an independent predictor of prognosis for patients with HNSCC, the Cox regression analysis was performed. The univariate Cox regression analysis performed on the entire cohort revealed that age, stage, T stage, and risk score were directly associated with the prognosis of HNSCC (*p* ≤ 0.001) (Fig. 6A). Additionally, the multivariate Cox regression analysis showed that the risk score was still an independent indicator in the prognosis of HNSCC (*p* < 0.001) (Fig. 6B). ROC curve analysis was used to evaluate the predictive ability of the risk model for overall survival (OS) in HNSCC patients. The risk score exhibited the highest AUC compared to other clinical and pathological features (Fig. 6C). Moreover, the risk score demonstrated good predictive performance (AUC = 0.722 for 1-year, 0.771 for 3-year, and 0.775 for 5-year survival) (Fig. 6D). In conclusion, these findings suggested that the risk score derived from the prognostic risk model based on mcrmRNA was an effective tool for assessing prognosis.

### Construction and validation of the nomogram

**Fig. 7:**
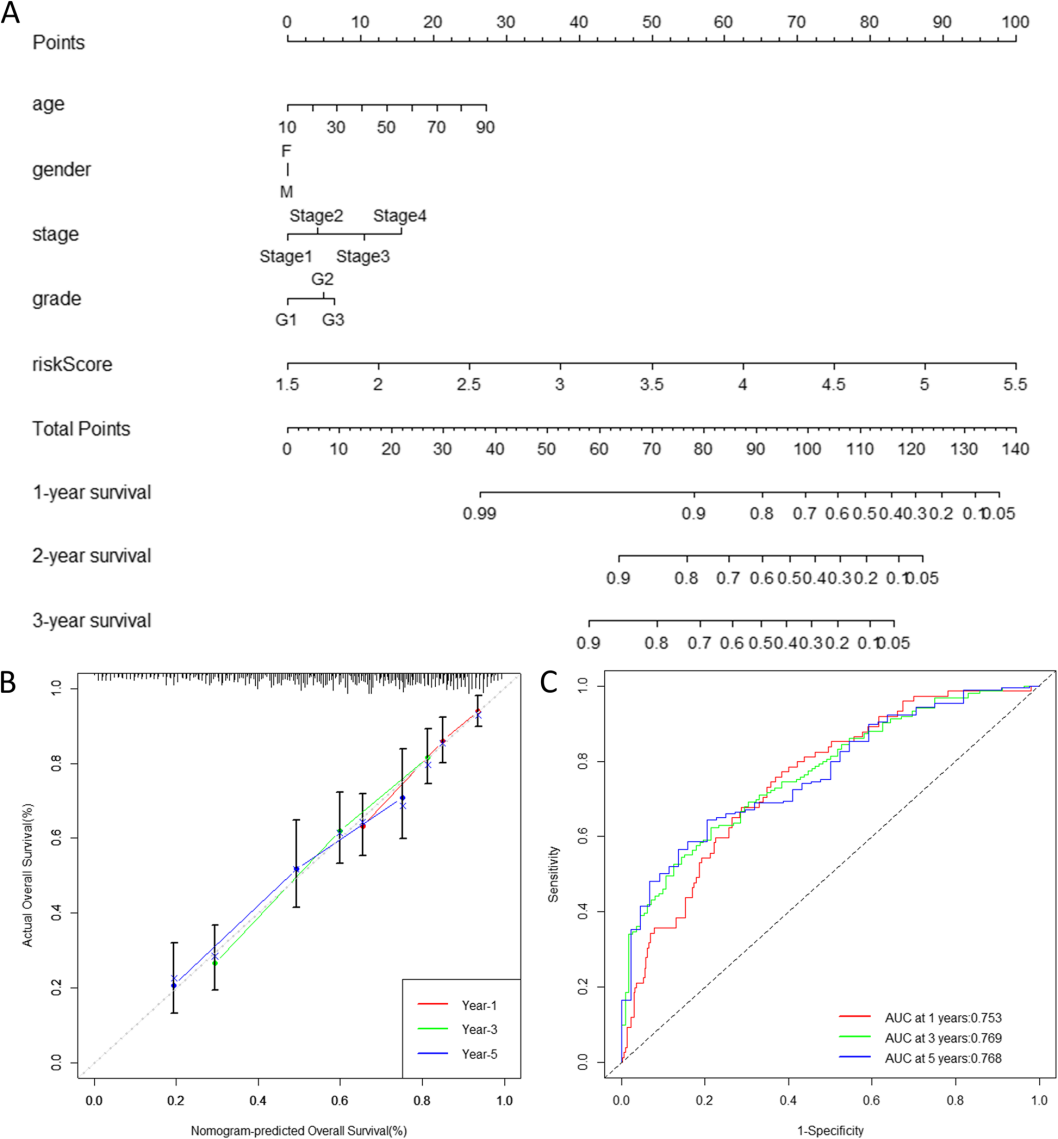
Construction of the nomogram incorporating clinical factors and the mcrmRNA prognostic risk model. **A** Nomogram for predicting the survival outcomes of HNSCC patients. **B** Calibration plots to assess the concordance between predicted and actual survival probabilities at 1, 3, and 5 years. **C** Time-dependent ROC curve for the nomogram

The nomogram is a statistical predictive model that creates a simple graphical representation by integrating multiple correlates and then generates numerical probabilities of clinical events. It has been used as an excellent tool for predicting the individual outcomes of patients with a wide range of cancers. To facilitate the application of the mcrmRNA prognostic risk model in clinical settings, a nomogram chart (Fig. 7A) was developed to integrate both the risk score and clinical pathological information. The calibration curve (Fig. 7B) demonstrated that the nomogram accurately predicted the probability of OS at 1, 3, and 5 years, which closely matched the observed OS. Additionally, the time-dependent ROC curve (Fig. 7C) indicated that the nomogram exhibited a good predictive ability for 1-year, 3-year, and 5-year survival (AUC = 0.753 for 1-year, 0.769 for 3-year, and 0.768 for 5-year survival). These findings provided strong evidence of the capability of the nomogram chart to predict the prognosis of HNSCC patients.

### Immune infiltration in the mcrmrna prognostic risk model

**Fig. 8:**
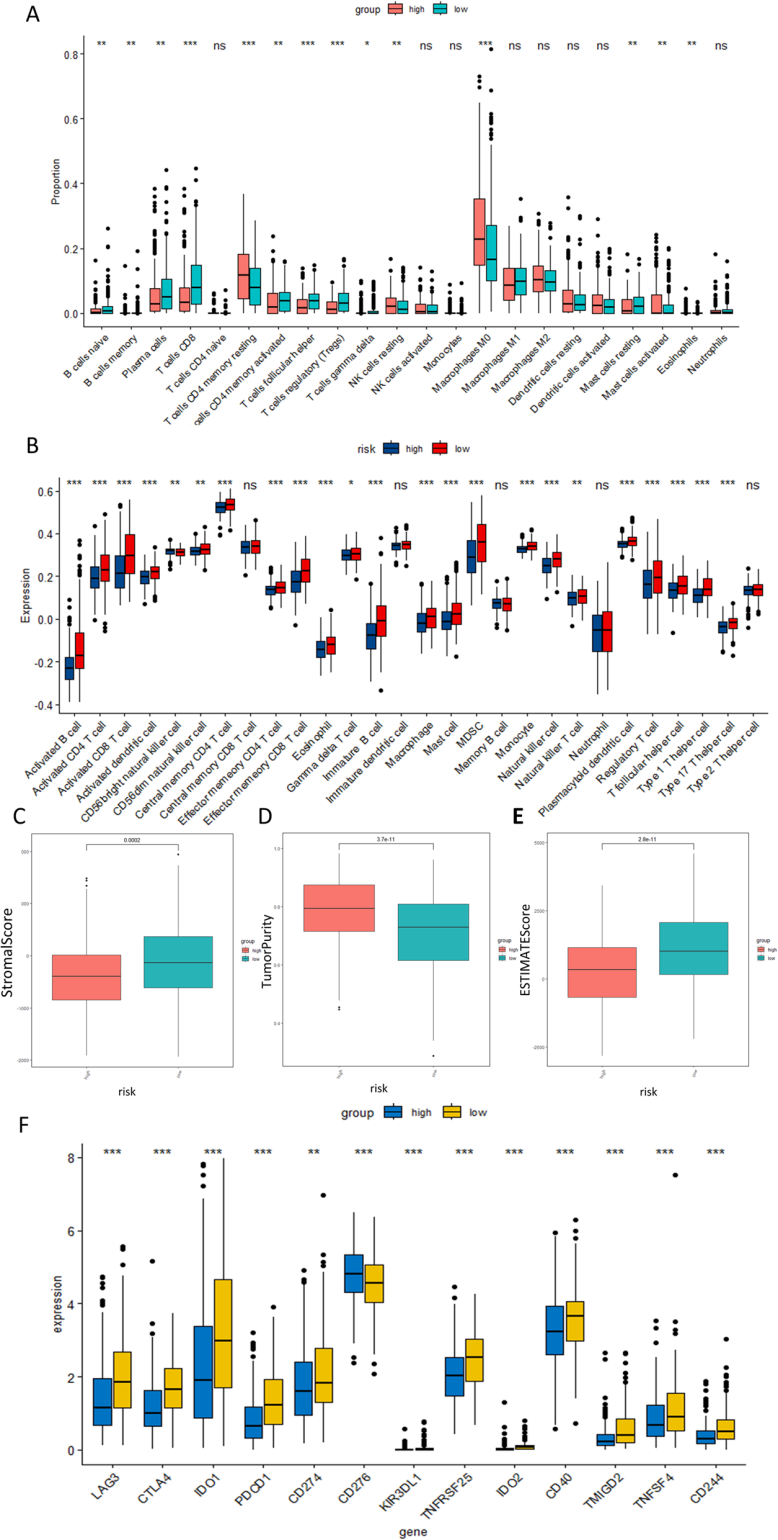
Immune infiltration characteristics based on the mcrmRNA prognostic risk model. **A** Immune cell infiltration of 22 immune cell types in high- and low-risk populations based on CIBERSORT analysis. **B** Differential analysis of 29 immune features between high- and low-risk populations based on ssGSEA analysis. **C** Differential analysis of stromal score, tumor purity, and immune score between high-risk and low-risk populations based on ESTIMATE analysis. **D** Boxplots comparing immune checkpoint genes between high- and low-risk groups of HNSCC patients

The tumor microenvironment (TME) consists of a large number of non-tumor cells, including immune cells and stromal cells, which strongly modulate cancer growth and invasion. To elucidate the relationship between immune infiltration and the high- and low-risk groups, we quantified the concentration fraction of 22 immune cells in the TME of each group. The results of the CIBERSORT and ssGSEA analyses (Fig. 8A, B) demonstrated higher enrichment of CD8^+^ T cells, CD4^+^ T cells, macrophages, plasma-like dendritic cells, and regulatory T cells (Tregs) in the low-risk group. Additionally, the ESITMATE analysis indicated that the low-risk group had a higher stromal score than the high-risk group (Fig. 8C, *p* < 0.001). Conversely, the high-risk group exhibited higher tumor purity than the low-risk group (Fig. 8D, *p* < 0.001), while the low-risk group had a higher immune score than the high-risk group (Fig. 8E, *p* < 0.001). Moreover, immune checkpoint blockade is an effective cancer immunotherapy. We further investigated expressions of immune checkpoint genes in the two risk groups to determine whether the prognostic model could serve as a potential marker to distinguish HNSCC patients suitable for immune checkpoint blockade therapy. The results revealed that the low-risk group displayed higher activity in immune checkpoint gene expression, including LAG3, CTLA4, IDO1, PDCD1, CD274, TNFRSF25, and IDO2 (Fig. 8F). These findings suggested that low-risk patients might exhibit increased responsiveness to immune therapy.

### Tumor mutation burden in the mcrmrna prognostic risk model

**Fig. 9:**
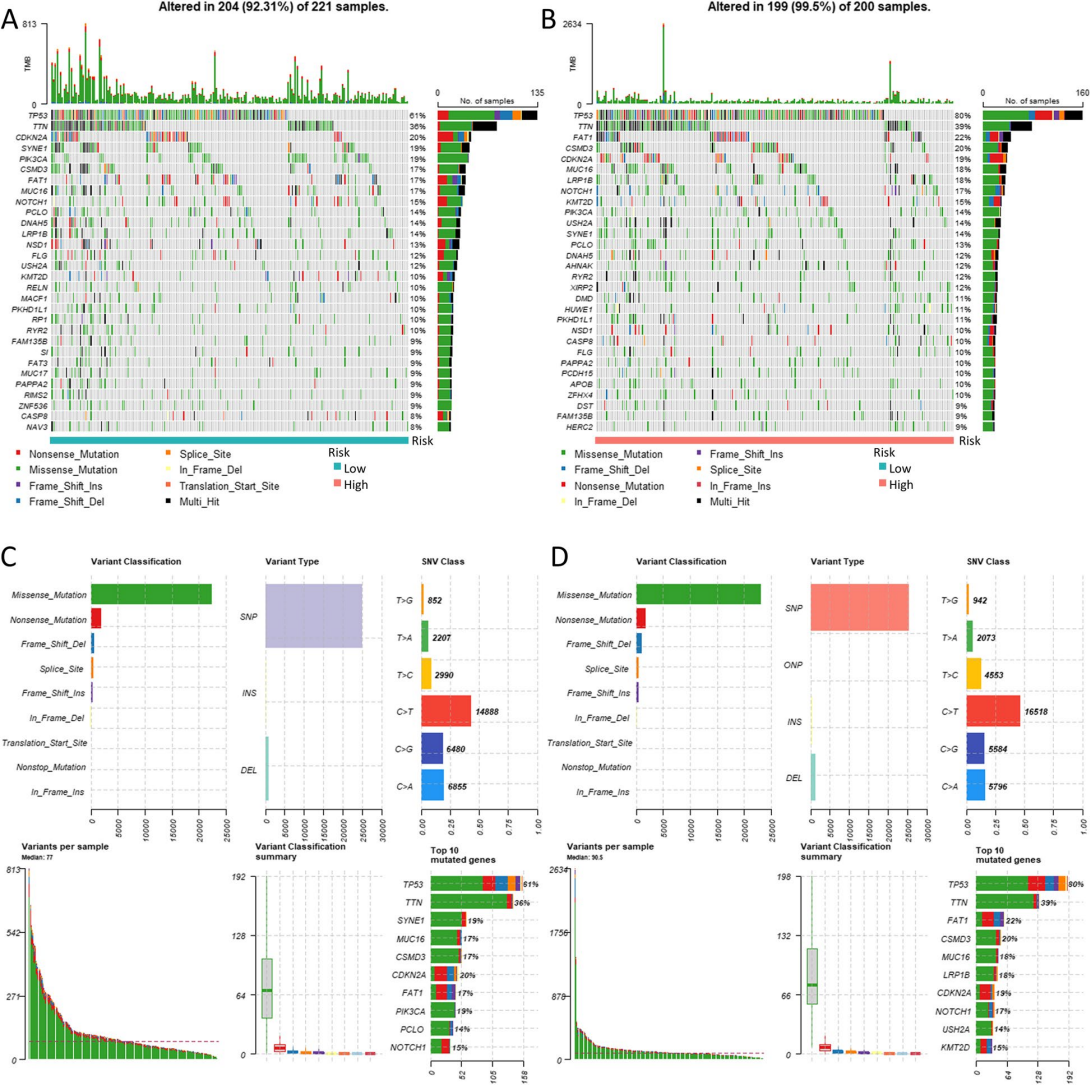
The analysis of somatic mutations in HNSCC patients. The waterfall plots (**A**, **B**) and the MAF (Mutation Annotation Format) summary plots (**C**, **D**) illustrate the somatic mutation profiles in the high- and low-risk groups of HNSCC patients

Additionally, we investigated mutation profiles of HNSCC patients in the high- and low-risk groups from the TCGA cohort. Waterfall plots showed the 20 most mutated genes in the two groups (Fig. 9A, B). The higher TMB was observed in the high-risk group. The high-risk group had TP53 (80%), TTN (39%), FAT1 (22%), CSMD3 (20%), and CDKN2A (19%) as the top five most frequently mutated genes (Fig. 9D). Conversely, in the low-risk group, TP53 (61%), TTN (36%), CDKN2A (20%), SYNE1 (19%), and PIK3CA (19%) were the genes with the highest mutation frequencies (Fig. 9C). These findings suggest that our model has the potential to identify patient candidates for immunotherapy and enhance therapeutic outcomes.

### The sensitivity of the two risk groups to clinical drugs

**Fig. 10:**
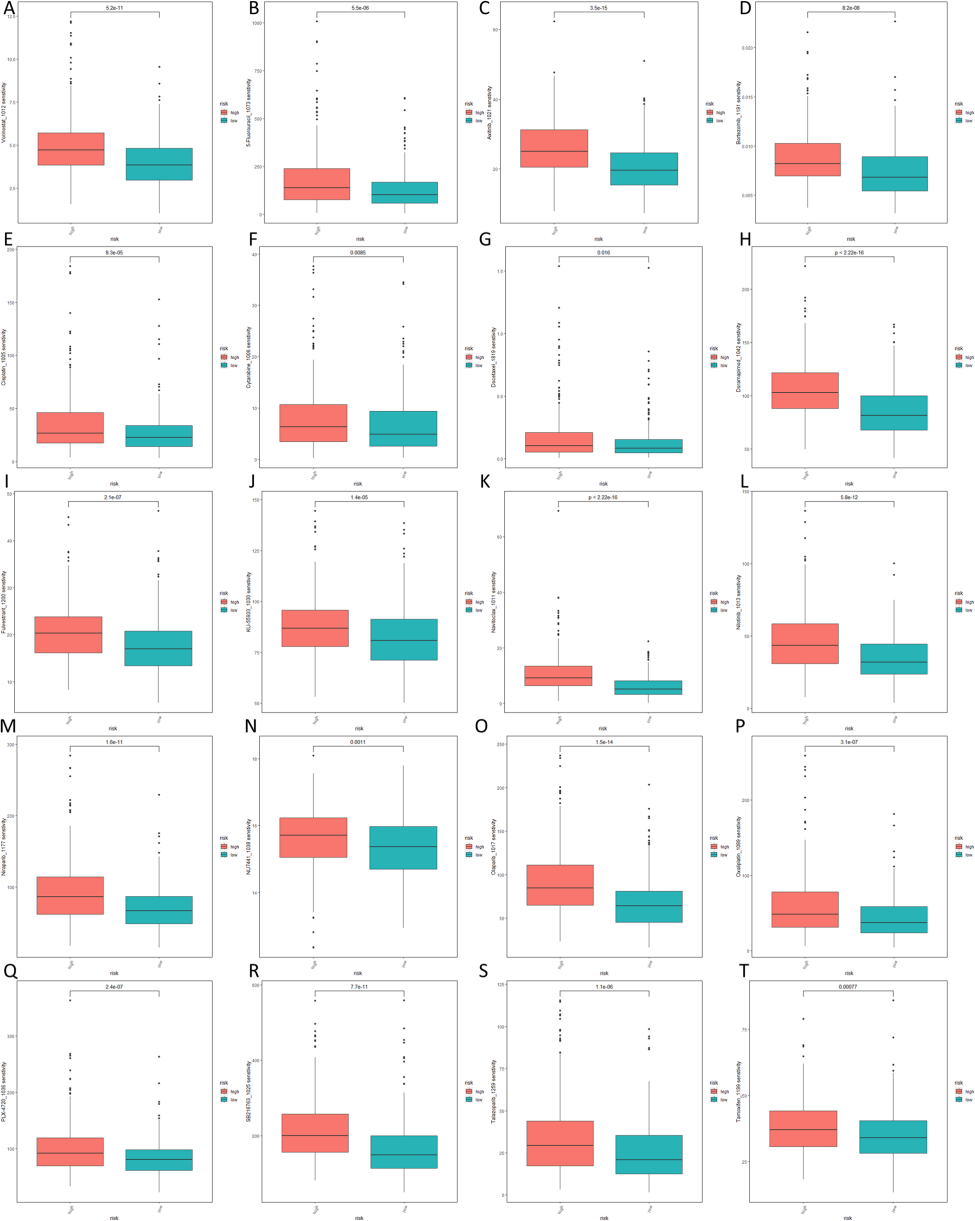
Drug sensitivity analysis. (**A**-**T**) Many therapeutic drugs exhibited higher IC50 values in HNSCC patients with high-risk scores compared to those with low-risk scores

Chemotherapy remains widely recognized as one of the primary and effective treatment approaches for cancer. Additionally, the risk model was utilized to calculate the IC50 value, a crucial parameter for evaluating the effectiveness of a drug and analyzing how a sample responds to treatment. In our study, several therapeutic agents had significantly higher IC50 values in patients at high risk for HNSCC (Fig. 10A-T). These drugs included Vorinostat_1012, 5-Fluorouracil_1073, Axitinib_1021, Bortezomib_1191, Cisplatin_1005, Cytarabine_1006, Docetaxel_1819, Doramapimod_1042, Fulvestrant_1200, KU-55933_1030, Navitoclax_1011, Nilotinib_1013, Niraparib_1177, NU7441_1038, Olaparib_1017, Oxaliplatin_1089, PLX-4720_1036, SB216763_1025, Talazoparib_1259, and Tamoxifen_1199.

### GO, KEGG, and GSEA pathway enrichment analysis

**Fig. 11:**
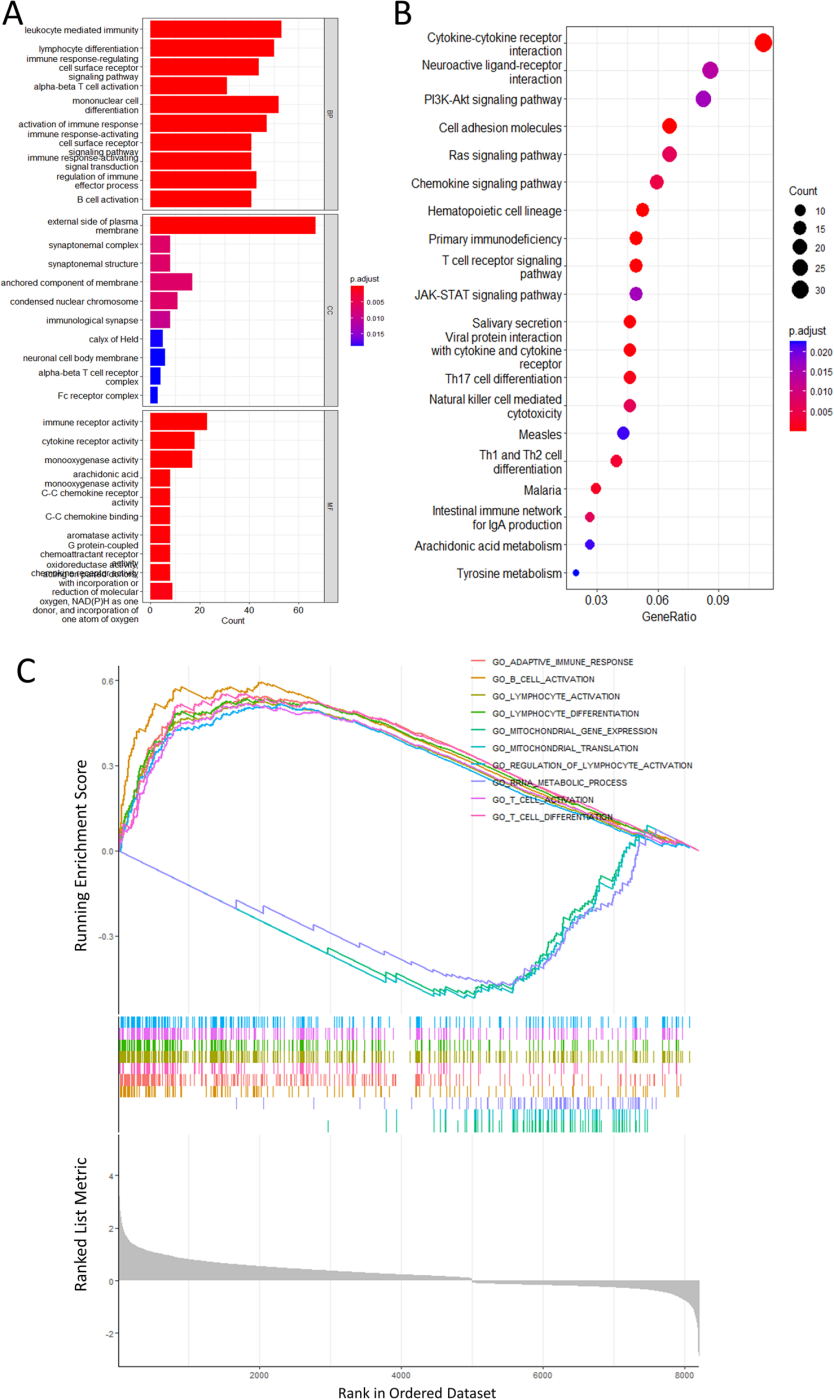
Biological function and pathway enrichment analysis of DEGs based on the mcrmRNA prognostic risk model (**A**) GO Enrichment Analysis. BP, biological process; CC, cellular component; and MF, molecular function. **B** KEGG Pathway Analysis. **C** GSEA Enrichment Analysis showing the activation status of biological pathways in the high- and low-risk groups

Based on transcriptomic data from the TCGA database, GO, KEGG, and GSEA enrichment analyses were used to further explore differences in biological functions and signaling pathways between high- and low-risk groups. Log2|FC|> 1 and FDR < 0.05 were selected as cutoff criteria for differentially expressed genes (DEGs) in the high- and low-risk groups. GO analysis (Fig. 11A) showed that DEGs were involved in biological processes (BP) such as leukocyte-mediated immunity, lymphocyte differentiation, immune response regulation, cell surface receptor signaling pathway, and α-βT cell activation. DEGs were enriched in cell components (CC) categories, including the extracellular side of the membrane, synaptic complex, α-βT cell receptor complex, synaptic membrane structure, and neuronal cell body membrane. Furthermore, DEGs were primarily associated with molecular function (MF) categories such as immune receptor activity, monooxygenase activity, and cytokine receptor activity. In KEGG analysis (Fig. 11B), these DEGs were enriched in cytokine-cytokine receptor interaction, neuroactive ligand-receptor interaction, phosphoinositide 3-kinase (PI3K)/AKT signaling pathway, cell adhesion molecules, RAS signaling pathway, and chemokine signaling pathway. The biological functions and signaling pathways enrichment between the two groups were validated using GSEA, and the GSEA enrichment plot (Fig. 11C) visualized the top 10 active pathways, demonstrating the enrichment of DEGs in immune-related pathways. In summary, the results of the functional enrichment analysis uncovered activated pathways and mechanisms that could be potentially involved in tumorigenesis and progression. This information offered valuable insights for assessing the prognosis of patients with HNSCC.

## Discussion

HNSCC ranks as the sixth most prevalent cancer globally, with Asia exhibiting the highest incidence rate. However, the intricate pathogenesis of HNSCC remains unclear and more research is needed. The proposal of cuproptosis and m6A provides a new theoretical foundation for tumor development and anti-tumor therapies. A study has identified a genetic model based on the association of cuproptosis to predict the prognosis of HNSCC [19]. The results indicate that this model demonstrates superior prognostic value compared to other clinical features such as the STAGE staging and tumor GRADE grading. In another study on the relationship between an RNA modification-associated factor model and HNSCC prognosis [20], the model achieved an AUC value of 0.652, slightly better than other clinical features, and time-dependent ROC curves of the model indicated AUC values of 0.652, 0.688, and 0.683 at years 1, 3, and 5, respectively. While these models show promise in clinical settings, they still lack high accuracy. Therefore, more accurate prognostic models are needed to predict the prognosis of HNSCC. This study combines cuproptosis and m6A, two biomarkers, to construct a prognostic model aimed at improving the accuracy of the prognostic model.

To elucidate the roles of m6A and cuproptosis in HNSCC and the potential link between them, our study first performed univariate Cox regression analysis, followed by correlation analysis of prognosis-related genes and cuproptosis as well as m6A, respectively. A 32 mcrmRNA model was successfully established by LASSO Cox regression analysis based on prognosis-related genes. These 32 mcrmRNA demonstrated significant correlations with both m6A and cuproptosis, with several of these genes already validated through in vitro and in vivo experiments. The loss of Cyclin-dependent kinase inhibitor 2A (CDKN2A) gene expression has been identified as a mechanism associated with oral squamous cell carcinoma [21]. CDKN2A, located on chromosome 9p21, encodes two distinct tumor suppressor proteins, p14ARF and p16INK4a, which play critical roles in regulating key pathways involved in tumor suppression, particularly the P53 pathway [22]. CDKN2A functions in tumorigenesis by controlling cell division, apoptosis, and maintaining cellular homeostasis through inhibition of cell cycle progression at the G1/S phase transition [23, 24]. The m6A modification is catalyzed by a methyltransferase complex composed of methyltransferase-like 3 (METTL3), methyltransferase-like 14 (METTL14), and Wilms tumor 1 associated protein (WTAP) [25]. The knockdown of METTL3 and METTL14 has been demonstrated to reduce mRNA m6A levels and downregulate the expression of tumor suppressor genes, including CDKN2A [26]. This decrease in mRNA m6A levels has been associated with enhanced in vitro growth and self-renewal of glioblastoma stem cells, along with an increase in the formation of brain tumors in vivo [27, 28]. Conversely, overexpression of METTL3 can elevate m6A levels in glioblastoma stem cells and suppress their growth. Moreover, studies have shown that CDKN2A sensitizes the cells to cuproptosis [10, 29]. Our investigation has recognized CDKN2A as a safeguarding gene in determining the prognosis of HNSCC patients. Given the strong correlation between CDKN2A, m6A, and cuproptosis, further exploration of CDKN2A's role in the intricate interplay of m6A and cuproptosis in HNSCC is warranted. Cytotoxic T lymphocyte-associated antigen 4 (CTLA4), functioning as a negative immune checkpoint, exhibits high expression levels in various solid tumors. CTLA4 primarily suppresses T cell responses by attenuating the signaling amplitude of co-stimulatory molecule CD28. The phenotype observed in CTLA4 knockout mice, characterized by lethal systemic immune hyperactivation, underscores the pivotal role of CTLA4 in dampening T cell activation and maintaining immunologic homeostasis [30]. Targeting CTLA4 has been reported to effectively reverse immunosuppression and improve outcomes in HNSCC patients by inhibiting myeloid-derived suppressor cell and M2 macrophage recruitment, while concurrently promoting T cell activation [31]. Protein Kinase C Eta (PRKCH), a member of the protein kinase C family, is involved in regulating both apoptosis and anti-apoptosis processes [32]. Studies have shown that PRKCH is linked to improved survival rates in individuals with head and neck cancer, which is consistent with the results of our research [33]. Matrix metalloproteinase-19 (MMP19) plays critical roles in various physiological and pathological processes, including inflammation, wound healing, and the progression of vascular [34]. Notably, MMP19, which is expressed in the tumor-invasive fronts, has been implicated in facilitating the invasiveness of HNSCC [35]. Thrombospondin 1 (THBS1), identified as an oncogene in oral squamous cell carcinoma (OSCC), acts as a tumor-specific extracellular matrix (ECM) protein induced by TGFB1 [36, 37]. THBS1 promotes cancer cell migration and stimulates matrix metalloproteinases (MMPs) through integrin signaling, facilitating OSCC invasion. Additionally, THBS1 mediates the PI3K/AKT signaling pathway to regulate OSCC cell proliferation, migration, and invasion [38]. While the roles of other genes in this risk model in HNSCC are not extensively studied, our research suggests that these genes play significant roles in HNSCC tumorigenesis and could be potential targets for cancer therapy. However, further investigation is necessary to fully comprehend their specific mechanisms and functions in HNSCC.

The risk scores calculated by the 32 mcrmRNA model can serve as independent prognostic indicators for the survival of HNSCC patients. The Kaplan–Meier analysis showed that a higher risk score was associated with a poorer prognosis. The ROC curve showed that the performance of our predictive model exhibited a more reliable and prominent performance compared to other clinical indicators. The risk model exhibited more favorable predictive ability with AUC values of 0.722, 0.771, and 0.775 for 1-year, 3-year, and 5-year survival, respectively. Moreover, various clinical features, including age, gender, tumor grade, TNM stage, and treatment, have been utilized in the construction of a nomogram chart for predicting the survival of HNSCC patients [39]. Thus, we established a brand-new nomogram chart that incorporates risk scores and clinical pathological information for HNSCC patients. The calibration curve exhibited a notable agreement between the predicted outcomes derived from the nomogram chart and the corresponding actual results. Collectively, these findings demonstrated the high accuracy and sensitivity displayed by our prognostic model. In comparison to existing prediction models, our prognostic risk score model combined two biomarkers that are critical to cancer, resulting in a more precise evaluation of the prognosis for patients with HNSCC. It established a foundation for further research on the specific roles of cuproptosis and m6A in HNSCC. Moreover, the mcrmRNA model possessed a notable advantage in facilitating the risk stratification of HNSCC patients, a critical aspect for patients and clinical practice. Tailoring treatments based on different risk levels not only improves patient prognosis but also optimizes the utilization of limited medical resources [40]. In conclusion, the mcrmRNA model exhibited higher reliability in predicting the prognosis of HNSCC patients than other clinical or pathologic variables.

The tumor immune microenvironment plays a pivotal role in the development and progression of HNSCC by promoting invasive tumor growth and treatment resistance, which subsequently negatively impacts prognosis [41, 42]. Within the immunosuppressive tumor microenvironment, HNSCC cells evade immune surveillance through various mechanisms [43]. To further investigate the potential connection between risk score and immune-related features, we conducted CIBERSORT and ssGSEA analyses to quantify immune cell infiltration in high- and low-risk groups. The results demonstrated enrichment of B cells naive, B cells memory, plasma cells, T cells CD8, T cells CD4 memory activated, T cells follicular helper, Tregs, T cells gamma delta, mast cells resting, and eosinophils in the low-risk group. Conversely, macrophages M0 and activated mast cells were enriched in the high-risk group. Macrophages play a crucial role in the tumor microenvironment, with a high presence of M0 macrophages being associated with a poor prognosis in early-stage lung cancer [44]. Moreover, activated mast cells induce neovascularization through the release of angiogenic factors and contribute to tumor aggressiveness by releasing various matrix metalloproteinases [45]. However, intratumor mast cells also exhibit a protective effect against prostate cancer recurrence and may serve as a prognostic biomarker following prostate cancer resection [46]. In patients with oral squamous cell carcinoma, tumor-draining lymph nodes (TDLNs) show a higher percentage of B cells compared to non-TDLNs, and are strongly linked to HNSCC progression [47]. However, our study revealed that patients in the low-risk group exhibit enrichment of naive B cells and memory B cells. This inconsistency may be attributed to the inherent constraints of the CIBERSORT algorithm, which has a tendency to either over- or under-estimate certain cell types systematically, despite demonstrating a relatively lower estimation bias. The ESTIMATE scoring results revealed inadequate immune efficacy in the high-risk group. In this study, the low-risk group exhibited abundant immune cell infiltration, including CD8^+^ T cells and Tregs, and showed more favorable prognosis outcomes. Previous research has demonstrated that a direct interaction between abundant tumor-infiltrating B-cells (TIL-B) and a higher density of B cells/CD8^+^ T cells leads to better prognoses for patients [48], and increased infiltration of CD8^+^ T cells has been closely associated with improved OS and local control (LRC) [42]. Tregs play a crucial role in maintaining a balance between self-immunity and immune suppression. Moreover, they have diverse functions within the TME, with a particular emphasis on inhibiting T cell activation [49]. Additionally, several studies have reported a correlation between Treg infiltration and improved OS and disease-free survival (DFS) in HNSCC patients [50, 51]. Discrepancies in immune cell infiltration between high- and low-risk groups may account for the varying prognoses observed in HNSCC patients. A notable correlation was found between immune infiltration, m6A modification, and cuproptosis. The m6A regulators are crucial in pathological and physiological immune cell infiltration and immune responses, essential for maintaining homeostasis and tumor immunosurveillance functions [52]. YTHDF1, a specific m6A regulator, enhances lysosomal cathepsin translation in dendritic cells, facilitating antigen presentation to CD8^+^ T cells [53]. This process impacts the cross-presentation of tumor neoantigens and the cross-priming of CD8^+^ T cells, aiding tumor immune evasion. Furthermore, METTL3 has been implicated in bladder cancer by promoting resistance to CD8^+^ T cell cytotoxic effects through the upregulation of programmed death ligand 1 (PD-L1) expression, underscoring the importance of m6A methylation in tumor immunity modulation [54]. Jin proposed that the m6A regulator ALKBH5 may suppress tumor progression within the immune microenvironment via the RIG-1/IFNA axis [55]. Moreover, Yi demonstrated the potential role of m6A in regulating the immune microenvironment of HNSCC in conjunction with the PI3K/AKT/mTOR signaling pathway [56]. High-mobility group box 1 (HMGB1), a common non-histone nuclear protein found in cells, is crucial for maintaining nucleosome structure and function, influencing gene transcription, DNA damage repair, and chromosomal rearrangement [57]. Studies have shown that in non-small cell lung cancer (NSCLC), cells undergoing cuproptosis release HMGB1, which subsequently binds to the advanced glycosylation end product-specific receptor (AGER) [58]. This interaction activates macrophages and triggers the production of inflammatory cytokines, initiating a cascade of immune responses. The cGMP-AMP synthase (cGAS)-stimulator of interferon genes (STING) pathway is a crucial component of innate immunity by responding to DNA triggers and orchestrating diverse immune responses that impact different stages of cancer development, including initiation and metastasis [59]. Cuproptosis has been shown to enhance cancer immunity by activating the cGAS-STING signaling pathway in clear cell renal cell carcinoma cells [60]. Moreover, in mouse tumor models, the combined use of cuproptosis inducers (elesclomol and CuCl2) with anti-PD-1 therapy synergistically increases levels of circulating CD8^+^ T cells. In summary, immune infiltration appears to be linked to m6A modification and cuproptosis in HNSCC, but further research is required to understand the specific mechanisms involved.

In addition, cancer cells evade immune surveillance and facilitate tumor progression through the modulation of immune checkpoint genes. As a result, immune checkpoint inhibitors have emerged as a promising approach to enhance treatment outcomes for cancer patients. Consequently, these inhibitors have become a vital component in the field of cancer treatment. We investigated the differential expression of common immune checkpoint genes between the high- and low-risk groups. The findings revealed an upregulation of CD276 in the high-risk group, while other immune checkpoints, including LAG3, CTLA4, IDO1, PDCD1, CD274, TNFRSF25, and IDO2, were upregulated in the low-risk group. These results suggested that low-risk patients might demonstrate enhanced responsiveness to immunotherapy. A recent study has demonstrated the significant involvement of CD276 in the proliferation, invasion, and migration processes of cancer cells [61]. HNSCC cells with high CD276 expression have a greater stemness capacity as well as metastatic ability, and in vivo experiments also showed that CD276 antibody monotherapy could effectively inhibit HNSCC growth and metastasis [62]. Our study suggests a potential therapeutic avenue for high risk HNSCC patients by targeting the immune checkpoint CD276. Moreover, the TMB has been confirmed as a promising predictive indicator of immunotherapy efficacy [63, 64]. For example, TMB serves as a unique and complementary biomarker for predicting the response to anti-programmed death 1 (anti-PD-1) therapy in HNSCC [65]. Therefore, we assessed the TMB status in high- and low-risk groups and found higher TMB in the high-risk group. These findings highlight the potential of our prognostic model in guiding treatment decisions regarding the use of immunotherapy. Specifically, the high-risk group exhibits a higher mutation rate of the P53 gene. The P53 gene is also strongly associated with cuproptosis. Recent studies have identified an association between the P53 gene and mutations in CDKN2A, a key gene of cuproptosis [66, 67]. Overall, cuproptosis is likely to have a significant influence on the effectiveness of immunotherapy in HNSCC, particularly through its involvement in the P53 pathway. Therefore, our prognostic model serves as a reliable biomarker for predicting the response to immunotherapy and as a guide for exploring the role of cuproptosis in HNSCC immunotherapy.

The sensitivity of common chemotherapeutic agents to HNSCC differed between the two risk groups. Patients with HNSCC in the high-risk group exhibited higher IC50 values for multiple chemotherapy drugs. Notably, patients in the high-risk group may exhibit resistance to various chemotherapeutic agents including 5-fluorouracil, cisplatin, and oxaliplatin, which are recommended chemotherapy drugs for HNSCC according to clinical practice guidelines [68]. Therefore, more comprehensive studies are needed to reverse resistance and improve patient prognosis. Currently, 5-fluorouracil is utilized in the treatment of multiple cancers and has demonstrated remarkable advancements in various tumor therapies with the aid of nanocarriers [69]. Platinum-based drugs like cisplatin, carboplatin, and oxaliplatin are extensively employed in chemotherapy to eliminate cancer [70, 71]. However, the clinical application of platinum-based drugs is severely limited due to their lack of selectivity, systemic toxicity, and development of drug resistance [72, 73]. The development of new platinum-based drugs and their targeted modifications undeniably holds great potential for enhancing current anticancer treatments [74]. Overall, this study indicates that HNSCC patients with high risk scores may have an increased susceptibility to chemotherapy resistance. These findings have important implications for guiding the treatment of HNSCC patients.

To further explore the differences between the high- and low-risk groups, GO, KEGG, and GSEA analyses were performed to explore biological functions and pathways associated with risk scores. The results of the GO analysis revealed that the DEGs between the high- and low-risk groups were enriched in various immune response pathways, including leukocyte-mediated immunity. This suggests that there may be differences in the immune responses against cancer and responses to therapy between the two groups. KEGG analysis revealed the involvement of DEGs in multiple signaling such as PI3K/AKT signaling pathway, RAS signaling pathway, and chemokine signaling pathway. The PI3K pathway offers a new direction for the development of PI3K inhibitors due to its vital role in cancer recurrence [75]. For instance, the tumor suppressor gene PER1 inhibits glycolysis-mediated cell proliferation by regulating PI3K stability and PI3K/AKT pathway dependence, thereby inhibiting oral squamous cell carcinoma progression [63]. These findings indicate that PER1 may serve as a valuable therapeutic target for oral squamous cell carcinoma. In addition, GSEA showed that the rRNA metabolism pathway associated with RNA processing was significantly different in the two groups. This implies that these m6A and cuproptosis-related genes of the prognostic model may play a role in influencing HNSCC at the protein synthesis level. Interestingly, significant enrichment was observed in several metabolic pathways between the two groups, including mitochondrial gene expression and mitochondrial translation. Mitochondria are critical in the development and progression of cancer as a source of energy and in regulating oncogenic signaling [76]. Therefore, targeted therapies against mitochondria have shown effectiveness. For example, a previous study has shown that Triptolide (TPL) induces apoptosis in HNSCC cells by inhibiting mitochondrial hexokinase [77]. Several studies have highlighted the significant role of melatonin in the treatment of HNSCC by regulating mitochondrial function [78, 79]. The utilization of GO, KEGG, and GSEA analyses in this study provides valuable insights into the role of m6A and cuproptosis in the progression of HNSCC. It is important to acknowledge the limitations of each analysis method, such as statistical threshold selection and pathway annotation quality [80]. Collectively, these findings indicate that the differences between the high- and low-risk groups are closely related to mitochondrial function and the TCA cycle, while the TCA cycle was identified as a direct target for the onset of cuproptosis. Further experimental and clinical studies are required to confirm these results.

## Conclusion

In summary, the risk model based on 32 mcrmRNA represents a valuable tool for guiding the individualized treatment and improving the prognosis of HNSCC patients. It also provides important insights into the potential mechanisms of cuproptosis and m6A in HNSCC. However, some limitations of our study need to be considered. First, there is a lack of sufficient HNSCC samples and clinical follow-up data within our institution to validate the prognostic prediction model thoroughly. Further research in the subsequent clinical phase is necessary. Moreover, the specific impact of the identified mcrmRNA on HNSCC cell death and the underlying mechanisms of m6A remain inconclusive. Consequently, conducting additional in vivo and in vitro experiments is imperative.

## Data Availability

Data were analyzed using TCGA database (https://portal.gdc.cancer.gov) and GEO database (https://www.ncbi.nlm.nih.gov/geo/). The original contributions presented in the study are included in the article, further inquiries can be directed to the corresponding author.

## Abbreviations

AUC: Area under the curve
BP: Biological processes
CC: Cell components
CDKN2A: Cyclin-dependent kinase inhibitor 2A
CRGs: Cuproptosis-related genes
CTLA4: Cytotoxic T lymphocyte-associated antigen 4
crmRNA: MRNAs specific to cuproptosis
DEGs: Differentially expressed genes
DFS: Disease-free survival
GO: Gene Ontology
GSEA: Gene Set Enrichment Analysis
GEO: Gene Expression Omnibus
HNSCC: Head and neck squamous cell carcinoma
KEGG: Kyoto Encyclopedia of Genes and Genomes
m6A: N6-methyladenosine
mcrmRNA: MRNA related to m6A and cuproptosis
METTL3: Methyltransferase-like 3
METTL14: Methyltransferase-like 14
MF: Molecular function
mrmRNA: MRNAs specific to m6A regulators
MMPs: Matrix metalloproteinases
MMP19: Matrix metalloproteinase-19
OSCC: Oral squamous cell carcinoma
OS: Overall survival
PCA: Principal Component Analysis
PD-1: Programmed death 1
PI3K: Phosphoinositide 3-kinase
PRKCH: Protein Kinase C Eta
ssGSEA: Single-sample Gene Set Enrichment Analysis
WTAP: Wilms tumor 1 associated protein
TCA: The tricarboxylic acid
TCGA: The Cancer Genome Atlas
TDLNs: Tumor-draining lymph nodes
THBS1: Thrombospondin 1
TMB: Tumor mutational burden
TME: The tumor microenvironment
TPL: Triptolide
Tregs: Regulatory T cells
